# Metabolic engineering of *Saccharomyces cerevisiae* for second-generation ethanol production from xylo-oligosaccharides and acetate

**DOI:** 10.1038/s41598-023-46293-8

**Published:** 2023-11-06

**Authors:** Dielle Pierotti Procópio, Jae Won Lee, Jonghyeok Shin, Robson Tramontina, Patrícia Felix Ávila, Lívia Beatriz Brenelli, Fabio Márcio Squina, André Damasio, Sarita Cândida Rabelo, Rosana Goldbeck, Telma Teixeira Franco, David Leak, Yong-Su Jin, Thiago Olitta Basso

**Affiliations:** 1 Department of Chemical Engineering, Escola Politécnica, Universidade de São Paulo (USP), São Paulo, SP 05508-010 Brazil; 2 DOE Center for Advanced Bioenergy and Bioproducts Innovation (CABER), University of Illinois at Urbana-Champaign, Urbana, IL 61801 USA; 3 Department of Food Science and Human Nutrition, University of Illinois at Urbana-Champaign (UIUC), Urbana, IL 61801 USA; 4 Department of Biochemistry and Tissue Biology, Institute of Biology, University of Campinas (UNICAMP), Campinas, SP 13083-862 Brazil; 5 Environment and Technological Processes Program, University of Sorocaba (UNISO), Sorocaba, SP 18023-000 Brazil; 6 School of Food Engineering, University of Campinas (UNICAMP), Campinas, SP 13083-862 Brazil; 7 Interdisciplinary Centre of Energy Planning, University of Campinas (UNICAMP), Campinas, SP 13083-896 Brazil; 8 Departament of Bioprocesses and Biotechnology, School of Agriculture, Sao Paulo State University (UNESP), Botucatu, SP 18618-687 Brazil; 9 School of Chemical Engineering, University of Campinas (UNICAMP), Campinas, SP 13083-852 Brazil; 10 Department of Biology and Biochemistry, University of Bath, Claverton Down, Bath, BA2 7AY UK; 11 Present Address: Departamento de Química Fundamental, Instituto de Química, Universidade de São Paulo), São Paulo, SP 05508-900 Brazil; 12 Present Address: Synthetic Biology & Bioengineering Research Center, Korea Research Institute of Bioscience and Biotechnology, Daejeon, 34141 Republic of Korea

**Keywords:** Industrial microbiology, Metabolic engineering

## Abstract

Simultaneous intracellular depolymerization of xylo-oligosaccharides (XOS) and acetate fermentation by engineered *Saccharomyces cerevisiae* offers significant potential for more cost-effective second-generation (2G) ethanol production. In the present work, the previously engineered *S. cerevisiae* strain, SR8A6S3, expressing enzymes for xylose assimilation along with an optimized route for acetate reduction, was used as the host for expressing two β-xylosidases, GH43-2 and GH43-7, and a xylodextrin transporter, CDT-2, from *Neurospora crassa*, yielding the engineered SR8A6S3-CDT-2-GH34-2/7 strain. Both β-xylosidases and the transporter were introduced by replacing two endogenous genes, *GRE3* and *SOR1*, that encode aldose reductase and sorbitol (xylitol) dehydrogenase, respectively, and catalyse steps in xylitol production. The engineered strain, SR8A6S3-CDT-2-GH34-2/7 (*sor1*Δ *gre3*Δ), produced ethanol through simultaneous XOS, xylose, and acetate co-utilization. The mutant strain produced 60% more ethanol and 12% less xylitol than the control strain when a hemicellulosic hydrolysate was used as a mono- and oligosaccharide source. Similarly, the ethanol yield was 84% higher for the engineered strain using hydrolysed xylan, compared with the parental strain. Xylan, a common polysaccharide in lignocellulosic residues, enables recombinant strains to outcompete contaminants in fermentation tanks, as XOS transport and breakdown occur intracellularly. Furthermore, acetic acid is a ubiquitous toxic component in lignocellulosic hydrolysates, deriving from hemicellulose and lignin breakdown. Therefore, the consumption of XOS, xylose, and acetate expands the capabilities of *S. cerevisiae* for utilization of all of the carbohydrate in lignocellulose, potentially increasing the efficiency of 2G biofuel production.

## Introduction

Fuel ethanol production from sugar cane is a major contributor to the ongoing transition from fossil to renewable fuels and chemicals^1^. To increase production, whilst restricting land use requirements, demands greater efficiency through utilising all of the fermentable carbohydrate in the sugar cane; including those present in bagasse and straw, which is composed of lignocellulose^2,3^. Successfully accessing and fermenting these other substrates would also develop routes for utilizing agricultural by-products, such as straw and forestry residues. Furthermore, their procurement costs are relatively low, as well as being an abundant non-food feedstock^4^. The lignocellulosic biofuel production process requires the deconstruction of biomass into fermentable sugars and the conversion of sugars into biofuels^5^. Hemicellulose and cellulose play vital roles as the primary constituents in the secondary layers of wood fibre’s cell wall^6^. Together with lignin and other minor components, like extractives and minerals, they form the well-known natural composition of lignocellulose biomass^6,7^. Hemicellulose is a diverse group of polysaccharides that makes up 15–35% of plant biomass^6^. Different plant species have varying hemicellulose content, with spruce having 18%^8^, birchwood with 19.7%^9^, corn stover containing 22.1%^9^, sugar cane bagasse comprising 28.6%^10^, willow containing 29.3%^9^, and switchgrass having 31.4%^11^ hemicellulose.

Due to the complex integration of cellulose, hemicellulose, and lignin in the structure of lignocellulose, harsh pre-treatment is required to access the carbohydrate polymers for enzymatic hydrolysis and fermentation; this can result in the production of by-products such as furans, organic acids, phenols and inorganic salts which can inhibit microbial metabolism^12–14^. Pre-treatment aims to reduce the crystallinity of cellulose, and partially degrade hemicellulose and lignin to increase the susceptibility of the biomass to enzymatic cocktails; which are necessary to breakdown polysaccharides into fermentable monomeric sugars^13,15,16^. However, during the degradation of hemicellulose and lignin, acetic acid production is unavoidable as hemicellulose and lignin are acetylated^17,18^. This product is toxic to yeast metabolism, and therefore reduces sugar fermentation efficiency and biofuel yield^13,19,20^.

Weak organic acids, such as acetic acid, can diffuse undissociated through the cell membrane and disperse inside the cell, releasing protons and lowering the internal pH value^13,21^. To overcome the inhibitory effect of acetic acid, Zhang et al.^22^ introduced an optimized route for acetate reduction, through the introduction and expression of the acetylating acetaldehyde dehydrogenase (AADH) and acetyl-CoA synthetase (ACS) activities into a xylose-fermenting *S. cerevisiae* strain, which produces recombinant NADPH-linked xylose reductase (XR) and NAD^+^-linked xylitol dehydrogenase (XDH), resulting in strain SR8A6S3. This strategy enabled efficient xylose fermentation with 29.7% higher ethanol yield and 70.7% lower by-product (xylitol and glycerol) production; when cultivated in YP medium supplemented with 20 g L^−1^ glucose, 80 g L^−1^ xylose, and 8 g L^−1^ acetate under strict anaerobic (anoxic) conditions. The reduction of acetate to ethanol serves as an electron sink to alleviate the redox cofactor imbalance resulting from XR and XDH activities^23^, with the NAD^+^ generated from the reductive metabolism of acetate being available for XDH activity; thus reducing the production of xylitol and glycerol. Therefore, this strategy can provide multiple benefits for the ethanol industry^22^.

Although SR8A6S3 can tolerate acetic acid present in lignocellulosic hydrolysates, many other inhibitory compounds are also released during the pre-treatment steps^13^. While less severe pre-treatment could be considered for achieving a lower concentration of inhibitors, a large amount of cellulolytic enzyme cocktails would still be required for converting cellulose and hemicellulose into monomeric sugars; presenting unsolved economic and logistical challenges for the industry^24–27^. One possible strategy to achieve economic 2G ethanol is to use genetically modified *S. cerevisiae* strains to transport and intracellularly utilize cellulose and hemicellulose-derived oligosaccharides. Such a microorganism might have a competitive advantage over other microorganisms, such as contaminating bacteria and wild *Saccharomyces* and non-*Saccharomyces* species, which are not able to metabolize oligosaccharides. Additionally, this process may require lower amounts of hemi/cellulolytic enzymes, which should translate into an affordable process^7^.

In a previous work, Li et al.^25^ expanded xylose utilization using an engineered *S. cerevisiae* strain, to incorporate the transport and intracellular hydrolysis of XOS to xylose monomers through the expression of: two β-xylosidases, GH43-2 and GH43-7, a XOS-transporter, and CDT-2, from *N. crassa* in a xylose-utilizing host strain. Both glycoside hydrolases (GH) catalyse the hydrolysis of 1,4-β-d-xylosidic linkages in xylan^28^. This new strain could produce more than 30 g L^−1^ of ethanol in 72 h of cultivation in an optimized minimum medium (oMM) supplemented with 4% xylose and 3% XOS; comprised of xylobiose (X2) and xylotriose (X3), and under anaerobic conditions.

**Figure 1:**
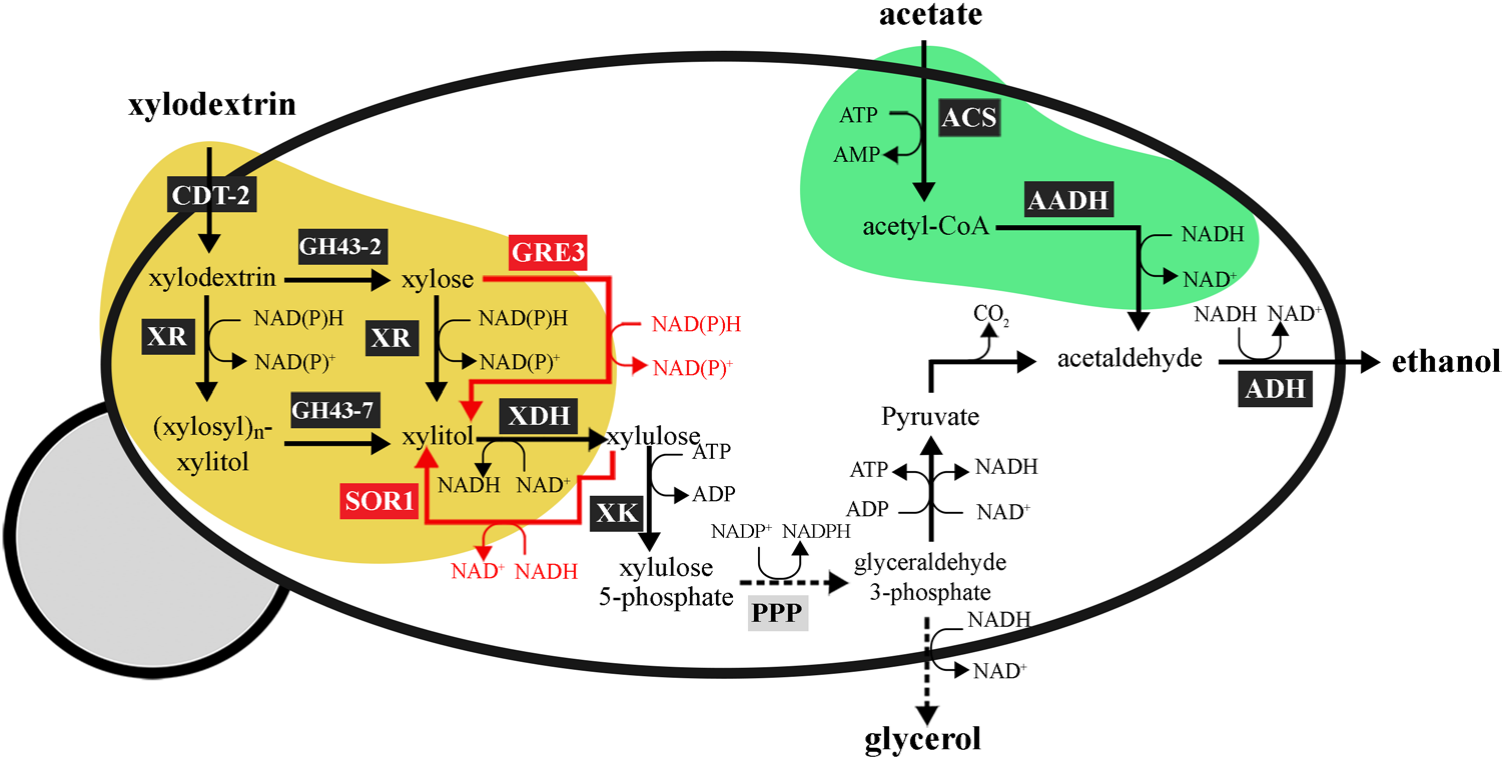
Expected routes of XOS metabolism after expression of the XOS-transporter (CTD-2) and beta-xylosidases (GH43-2 and GH43-7) from *N. crassa* in SR8A6S3*,* including xylose metabolism by xylose reductase (XR) and xylitol dehydrogenase (XDH) from *S. stipitis*. The surplus NADH produced during xylose fermentation can be exploited to detoxify acetate, reducing it to ethanol through the exogenous acetate reduction pathway, involving conversion of acetate into acetyl-CoA by ACS, production of acetaldehyde from the acetyl-CoA by AADH, and ethanol production from acetaldehyde through the action of alcohol dehydrogenase (*ADH*). Adapted from^22^.

In this work, we used the SR8A6S3 strain as a platform for the construction of a yeast strain able to ferment XOS, xylose, and acetate into ethanol (Fig. 1). Genes encoding the XOS-transporter (CDT-2) and both of the β-xylosidases (GH43-2 and GH43-7) from *N. crassa* were integrated into the SR8A6S3 genome (highlighted in the yellow area in Fig. 1). First, a high expression cassette for CDT-2 expression was integrated into the sorbitol (xylitol) dehydrogenase *locus*, encoded by gene *sor1* gene, through the locus-specific CAS-9-based integration system^29^. Then, both GH43-2 and GH43-7, under the control of the *GAP* and *CCW12* promoters, respectively, were integrated into the aldose reductase encoded by the *gre3* gene using the same locus-specific integration tool. The resulting disruption of *GRE3* and *SOR1* was designed to mitigate xylitol production and divert more carbon towards ethanol production in the recombinant strain^30–32^. Conversion of hemicellulose-derived residues into industrial products, such as 2G ethanol, can contribute to the progress of global warming mitigation^33^.

## Results and discussion

### Cas9- based integration of CDT-2 expression cassette into the *SOR1* locus

**Figure 2:**
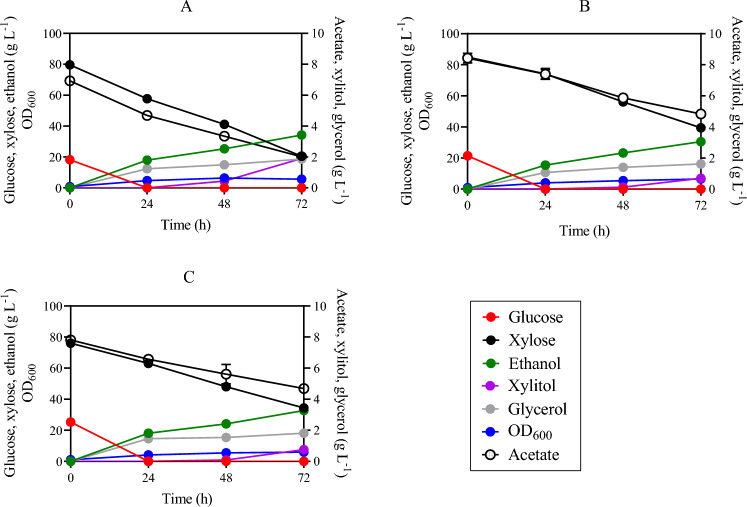
Fermentation profiles of SR8A6S3 (**A**), SR8A6S3-CDT-2 (**B**), and SR8A6S3-CDT-2-GH43-2/7 (**C**) when fermenting YP supplemented with 20 g L^−1^ glucose, 80 g L^−1^ xylose, and 8 g L^−1^ acetate under strict anaerobic conditions. Data are presented as mean values and standard deviations of three independent biological replicates.

**Figure 3:**
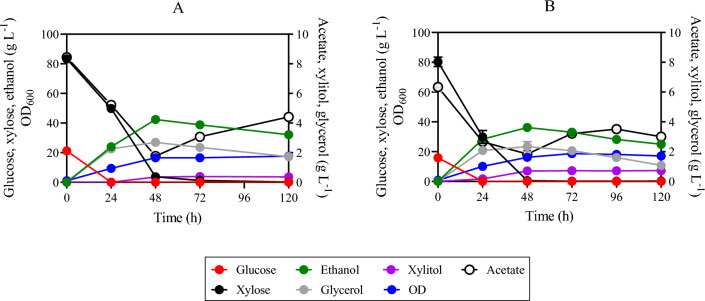
Fermentation profiles of SR8A6S3-CDT-2 (**A**), and SR8A6S3 (**B**) when fermenting 20 g L^−1^ glucose, 80 g L^−1^ xylose, and 8 g L^−1^ acetate under micro-aerobic conditions. Data are presented as mean values and standard deviations of three independent biological replicates.

Although xylitol has a variety of uses in the food, cosmetic, nutraceutical, and pharmaceutical industries^34^, this metabolite may face competition from an available carbon source, reducing the efficiency of ethanol production. *S. cerevisiae* strains possess genes that encode enzymes capable of xylose reduction, such as *GRE3*, *GCY1*, *YPR1*, *YDL124W*, *YJR096W*, and xylitol oxidation such as *SOR1*, *SOR2, XYL2*, *XDH1*; which can result in xylitol formation during xylose fermentation^35^. To reduce xylitol production and divert the carbon towards ethanol production, two distinct genes, namely *GRE3* and *SOR1*, were selected for knockout. First, *SOR1* was replaced by a CDT-2 expression cassette in the genomic DNA of strain SR8A6S3, yielding SR8A6S3-CDT-2 (*sor1*Δ). The required integration of the CDT-2 cassette was confirmed through PCR analysis. Colony PCR was performed directly from 27 colonies of the positive-control plate (Additional file 1; Fig. S1). Once the desired integration was confirmed, both the SR8A6S3-CDT-2 and SR8A6S3 strains were compared in anaerobic and micro-aerobic batch cultures (Figs. 2A, B and 3) in YPDXA containing: 20 g L^−1^ glucose, 80 g L^−1^ xylose, and 8 g L^−1^ acetate, with an initial OD_600_ of 1.

Deletion of *SOR1* led to a reduced rate of xylose and acetate consumption under both anaerobic and micro-aerobic conditions (Figs. 2A, B and 3). Under anaerobic batch cultivation, 75% of the initial xylose was consumed by the SR8A6S3 strain, while SR8A6S3-CDT-2 was only able to consume 53% of the original concentration within 72 h (Additional file 2: Fig. 2A). The xylose consumption rate of SR8A6S3 was also higher after 24 h of anaerobic cultivation in comparison to SR8A6S3-CDT-2 (Table 1).

**Table 1:** Fermentation profiles of SR8A6S3-CDT-2-GH43-2/7, SR8A6S3-CDT-2, and SR8A6S3 in YP media containing 20 g L^−1^ glucose, 80 g L^−1^ xylose, and 8 g L^−1^ acetate under anaerobic conditions.

	At 24 h				At 72 h					
	*r*_xylose_	*r*_xylose_*	*P*_xylitol_	*P*_ethanol_	*r*_xylose_	*r*_xylose_*	*P*_xylitol_	*P*_ethanol_	Y_ethanol_	Y_xylitol_
SR8A6S3-CDT-2-GH43-2/7	0.54 ± 0.03	0.15 ± 0.01	0.00 ± 0.00	0.77 ± 0.05	0.57 ± 0.01	0.10 ± 0.00	0.01 ± 0.00	0.45 ± 0.00	0.47 ± 0.01	0.018. ± 0.000
SR8A6S3-CDT-2	0.37 ± 0.13	0.10 ± 0.03	0.00 ± 0.00	0.62 ± 0.06	0.63 ± 0.03	0.10 ± 0.02	0.01 ± 0.00	0.41 ± 0.01	0.44 ± 0.01	0.015. ± 0.001
SR8A6S3	0.94 ± 0.06	0.19 ± 0.01	0.00 ± 0.00	0.74 ± 0.00	0.83 ± 0.02	0.14 ± 0.00	0.03 ± 0.00	0.47 ± 0.00	0.42 ± 0.00	0.032 ± 0.0000

Parameters: *r*_xylose_, xylose consumption rate (g L^−1^ h^−1^); *r*_xylose_*, specific xylose consumption rate (g L^−1^ OD^−1^ h^−1^); *P*_xylitol_, volumetric xylitol productivity (g L^−1^ h^−1^); *P*_ethanol_, volumetric ethanol productivity (g L^−1^ h^−1^); Y_ethanol_, ethanol yield (g g_consumed carbon source_^−1^); Y_xylitol_, xylitol yield (g g_consumed carbon source_^−1^).

Concerning acetate metabolism, the control strain consumed 71% of the initial acetate in the medium, while SR8A6S3-CDT-2 consumed only 43% in 72 h of cultivation (Fig. 2A, B, and Additional file 2: Fig. S2E). For glucose metabolism, no difference was observed between the two strains (Fig. 2). However, despite the greater consumption of xylose and acetate by the control strain (83.08 ± 1.46 versus 70.58 ± 2.91 g L^−1^), SR8A6S3-CDT-2 had a slightly higher ethanol yield (Table 1) and produced 66% less xylitol and 12% less glycerol as a by-product (Additional file 2: Fig. S2). We observed that glycerol was primarily coming from glucose for both cultivations. A great amount of total glycerol was produced after 24 h of cultivation, 66% for SR8A6S3 and 60% for SR8A6S3-CDT-2. Considering these results, it is possible to conclude that in the control strain cultivation, the carbon source was channelled towards metabolites whose pathways allowed for the balance of redox cofactors, such as xylitol and glycerol. Thereby, *SOR1* is responsible for a significant amount of xylitol production, but *sor1*Δ primarily slows down xylose metabolism. However, *sor1*Δ enabled the engineered strain to drive more carbon toward the desired product (ethanol). Presumably, this is because the NADH / NAD^+^ balance changed, while the ethanol yield had marginally increased via the pyruvate decarboxylase route as, relative to xylose, acetate metabolism is proportionally lower when the two strains are compared.

Other metabolites were also measured to compare the fermentation profiles of SR8A6S3-CDT-2 and SR8A6S3 (Additional file 2: Fig. S2). Elimination of xylitol production through *sor1*Δ increases the availability of intracellular NADH, which enabled the recombinant cell to produce more ethanol per gram of consumed sugar (ethanol yield). Deletion of the *sor1* gene does not eliminate xylitol production, since other genes encode enzymes capable of xylose reduction or xylitol oxidation, resulting in xylitol production. However, under strict anaerobic cultivation, the xylitol amount was reduced from 1.9 to 0.69 g L^−1^, when comparing the parental SR8A6S3 and recombinant SR8A6S3-CDT-2 strains, respectively (Additional file 2: Fig. S2C). In principle, NAD^+^ should be available to drive the xylitol to xylulose reaction.

Under strict anaerobic conditions, ethanol is the most important primary metabolite produced, in terms of re-oxidation of excess NADH and redox balancing, followed by the production of glycerol^36^, which is important to support xylulose production from xylitol. When oxygen is available in the flask, redox balancing of NADH/NAD^+^ can also occur through the electron transport chain, which should result in less xylitol accumulation in the medium. We corroborated this hypothesis during batch cultivations under micro-aerobic conditions, where lower xylitol production was observed for both strains (Fig. 3 and Additional file 3: S3C). Micro-aerobic batch fermentations were performed in complex YP media supplemented with 20 g L^−1^ glucose, 80 g L^−1^ xylose, and 8 g L^−1^ acetate with an initial OD_600_ of 1 (Fig. 3 and Additional file 3: S3).

Under micro-aerobic conditions the ethanol yields of SR8A6S3-CDT-2 and SR8A6S3 were 0.39 g_ethanol_ g_consumed sugars_^−1^ and 0.37 g_ethanol_ g_consumed sugars_^−1^, respectively. As expected, xylitol yield was lower in SR8A6S3-CDT-2 than in SR8A6S3, 0.004 g_xylitol_ g_consumed xylose_^−1^ against 0.009 g_xylitol_ g_consumed xylose_^−1^, respectively. Under 48 h of cultivation, SR8A6S3-CDT-2 consumed 79% of the initial acetate concentration, whereas SR8A6S3 consumed slightly lower amounts with 73%. After 48 h, however, both strains oxidised the ethanol back into acetate (Fig. 3).

### Cas9-based integration of a GH43-2_GH43-7 expression cassette into the *GRE3 locus*

*GRE3* is an important xylose-reducing enzyme expressed by *S. cerevisiae* strains, the deletion of which decreases xylitol formation^32^. Therefore, to further decrease carbon being diverted towards xylitol formation, a cassette for GH43-2 and GH43-7 high expression was integrated into the *GRE3* locus of SR8A6S3-CDT_2_ using a CAS-9-based system^29^, yielding the SR8A6S3-CDT-2-GH43-2/7 strain (*sor1*Δ *gre3*Δ). The desired integration of the GH43-2/7 sequence cassette into SR8A6S3-CDT-2-GH43-2/7 was confirmed through colony PCR performed on 7 colonies from the positive-control plate (Additional file 4: Fig. S4).

To investigate the engineered strain, a YP-based medium was used to cultivate SR8A6S3-CDT-2-GH43-2/7 and measure xylose and acetate fermentation performance; in comparison to SR8A6S3-CDT-2 and their parental strain, SR8A6S3. Anaerobic batch cultivation was carried out for xylose and acetate consumption evaluation; and ethanol and xylitol production in high sugar content media (20 g L^−1^ glucose, 80 g L^−1^ xylose, and 8 g L^−1^ acetate), with an initial OD_600_ of 1 (Fig. 2 and Additional file 5: S5).

In the first 24 h of cultivation, SR8A6S3-CDT-2-GH43-2/7 had an increased xylose consumption profile, compared with the SR8A6S3-CDT-2 strain. The latest engineered strain consumed 13.65 ± 0.53 g L^−1^ of xylose, which represents 18% of the initial xylose concentration; and the immediate parent consumed 10.73 ± 0.53 g L^−1^ (15% of the initial xylose concentration). During the same period, the acetate consumption profile was slightly higher for SR8A6S3-CDT-2-GH43-2/7 than SR8A6S3-CDT-2, 17% against 15% of the initial acetate concentration, respectively (Fig. 2B and C). Following a similar trend, the glycerol production profile within the first 24 h was higher for SR8A6S3-CDT-2-GH43-2/7 than for the SR8A6S3-CDT-2 strain. The former produced 1.47 ± 0.04 g L^−1^, and the latter achieved 0.98 ± 0.13 g L^−1^ of glycerol. Conversely, within 24 and 72 h of cultivation, SR8A6S3-CDT-2-GH43-2/7 consumed lesser amounts of xylose and acetate than its immediate parent strain, 28.84 ± 0.66 g L^−1^ against 34.61 ± 3.45 g L^−1^ for xylose, and 1.81 ± 0.14 g L^−1^ against 2.38 ± 0.14 g L^−1^ for acetate, respectively; as well as produced lesser amounts of glycerol, 0.34 ± 0.04 g L^−1^ against 0.66 ± 0.12 g L^−1^, respectively for SR8A6S3-CDT-2-GH43-2/7 and SR8A6S3-CDT-2 (Fig. 2B and C). The change in the xylose, acetate, and glycerol profile for both strains in the first 24 h of cultivation and after, is presumed to be because of the change in the NADH / NAD^+^ balance. Deletion of *GRE3* and increased production of glycerol (within 24 h of cultivation) might result in the higher availability of cofactors required for xylose metabolism (Additional file 6: Fig. S6), which reflected a better xylose consumption profile for SR8A6S3-CDT-2-GH43-2/7 in the first 24 h of cultivation. After the depletion of glucose, the glycerol production profile decreased for both strains (Fig. 2B and C), however, *gre3*Δ slows down xylose metabolism.

Instead, compared with SR8A6S3 (Fig. 2A and B), the latest engineered strain had impaired xylose and acetate consumption profiles at all times during cultivation. Within the first 24 h of cultivation, SR8A6S3 consumed 22.53 ± 1.24 g L^−1^ of xylose, which represents 28% of the initial concentration, and 2.28 ± 0.10 g L^−1^ of acetate. The SR8A6S3-CDT-2-GH43-2/7 strain after 72 h consumed only 56% and 40% of the initial xylose and acetate concentration, respectively. Although, intriguingly, the rate of xylose consumption was marginally higher than the parent strains after 24 h. However, SR8A6S3-CDT_2_-GH43_2/7_ had a slightly higher ethanol yield compared to both SR8A6S3-CDT_2_ and SR8A6S3 (Table 1). Therefore, although xylitol production after 72 h was similar for SR8A6S3-CDT-2-GH43-2/7 and SR8A6S3-CDT-2, the deletion of both *sor1*Δ and *gre3*Δ, should have increased the availability of reduced cofactors, and enabled cells to produce more ethanol per gram of consumed sugar (ethanol yield) than just the *sor1*Δ single deletion (Additional file 6: Fig. S6). Moreover, the biomass production profile, which was analysed by the measurement of OD_600_, was similar until 24 h for SR8A6S3-CDT-2-GH43-2/7 and SR8A6S3-CDT-2, but was lower for the reference strain (SR8A6S3). The double-engineered strain and its immediate parent (SR8A6S3-CDT-2) presented an increase in biomass content of 2.95 ± 0.01 and 2.81 ± 0.01, representing an increase of 376% and 384% of OD_600_ within the first 24 h of cultivation. In the meantime, SR8A6S3 achieved a growth of 584% of initial cell concentration, and achieved an OD_600_ of 3.90 ± 0.03 at 24 h of cultivation. The *sor1*Δ decreased the xylose consumption rate, while *gre3*Δ increased the rate at 24 h, but was still lower than SR8A6S3 (Table 1).

Several studies have reported that the deletion of the *gre3*Δ plus *sor1*Δ genes can enhance xylose fermentation in engineered yeast strains. For instance, Wenger et al.^35^ screened a large number of *S. cerevisiae* strains from wild, industrial, and laboratory backgrounds to determine the xylose-positive phenotype. Of 647 studied strains, some wine strains appeared to be able to grow modestly on xylose. Through the application of high-throughput sequencing to bulk segregant analysis, they were able to identify a novel *XDH* gene, homologous to *SOR1* (which was called *XDH1*), responsible for this phenotype. Next, the authors performed a comprehensive analysis of the involvement of the genes *GCY1, GRE3, YDL124W, YJR096W, YPR1, SOR1, SOR2, XDH1, XYL2,* and *XKS1* in the *XDH1* background strain (which has a xylose-positive phenotype) through single or combined deletion of the target genes. Single deletion of putative xylitol dehydrogenases (*SOR1*, *SOR2* and *XYL2*) increased the xylose utilization rate relative to the positive control; this phenotype was further enhanced when all three genes were deleted (*sor1*Δ *sor2*Δ *xyl2*Δ)^35^.

To assess the effect of endogenous xylitol-assimilating pathway genes on the xylitol production profile by an engineered *S. cerevisiae* industrial strain, CK17, overexpressing *Candida tropicalis XYL1* (encoding xylose reductase) in both batch and fed-batch fermentation with xylose and glucose as carbon sources. Yang et al.^37^ performed single deletion of the following genes: *XYL2* (yielding the strain CK17Δ*xyl2*), *SOR1*/*SOR2* (yielding the strain CK17Δ*sor*), and *XKS1* (yielding the strain CK17Δ*xks1*)^37^. According to the authors, the mutant *sorΔ* had a reduced xylose consumption rate (12.4%) and xylitol production rate (4.7%), compared with its parental CK17 strain, which is consistent with our findings for SR8A6S3-CDT-2. The strain, CK17Δ*xks1*, had the highest xylose consumption (0.65 g L^−1^ h^−1^) and xylitol production rate (0.644 g L^−1^ h^−1^), while the control strain consumed xylose and xylitol at 0.598 g L^−1^ h^−1^ and 0.549 g L^−1^ h^−1^, respectively^37^.

Träff et al.^32^ conducted a study where they deleted the *GRE3* gene to enhance xylose metabolism in *S. cerevisiae* CEN.PK2-1C, which expressed the xylose isomerase encoding gene *xylA* sourced from *Thermus thermophilus*^32^. The recombinant *gre3*Δ strains produced less xylitol than the parental strain^32^. According to the authors, deletion of *GRE3* in *S. cerevisiae* decreased xylitol formation two- to threefold but not completely, as xylitol may also be formed by the products of other genes, such as *XDH* (homologous to *SOR1* gene), or through the reduction of xylulose or putative XR enzyme^35,38,39^. Similarly, in the construction of a *S. cerevisiae* strain expressing the isomerase pathway (*xylA*) from the anaerobic fungus *Orpinomyces* sp. (GenBank No. MK335957), several genetic modifications were made. Specifically, the *gre3*Δ, *sor1*Δ deletions were introduced along with the overexpression of *XYL3* and *TAL1* genes. These genetic modifications aimed to reduce xylitol accumulation and increase the growth rate^30^.

On the other hand, overexpression of the endogenous genes *GRE3* and *XYL2* under endogenous promoters, coding for nonspecific aldose reductase and xylitol dehydrogenase, respectively, enhanced the growth of *S. cerevisiae* on xylose in the presence of glucose in aerobic shake flask cultivation^31^. However, significantly more xylitol was formed by the CEN.PK2 strain overexpressing the *S. cerevisiae* enzymes in comparison to the strain that carries XR and XDH from *S. stipitis*. Furthermore, transcriptional analysis of xylose and glucose grown cultures shows that the expression of *SOR1*, which encodes sorbitol dehydrogenase, was elevated in transformed cultures. Therefore, the presence of xylose resulted in higher XDH activity and induced the expression of the *SOR1* gene, which also has XDH activity^31^.

*GRE3* and *SOR1* genes were considered for improving xylose fermentation based on these previous studies. In some of them, *sor1*Δ increased xylose utilization, and *gre3*Δ plus *sor1*Δ decreased xylitol accumulation. Similarly, we have observed that *gre3*Δ plus *sor1*Δ in *S. cerevisiae* SR8A6S3 decreased xylitol formation (Table 1). However, in contrast, *sor1*Δ alone did not increase the xylose consumption rate of SR8A6S3 (Table 1) as reported by^35^.

### GH43 beta-xylosidases are intracellularly active

**Figure 4:**
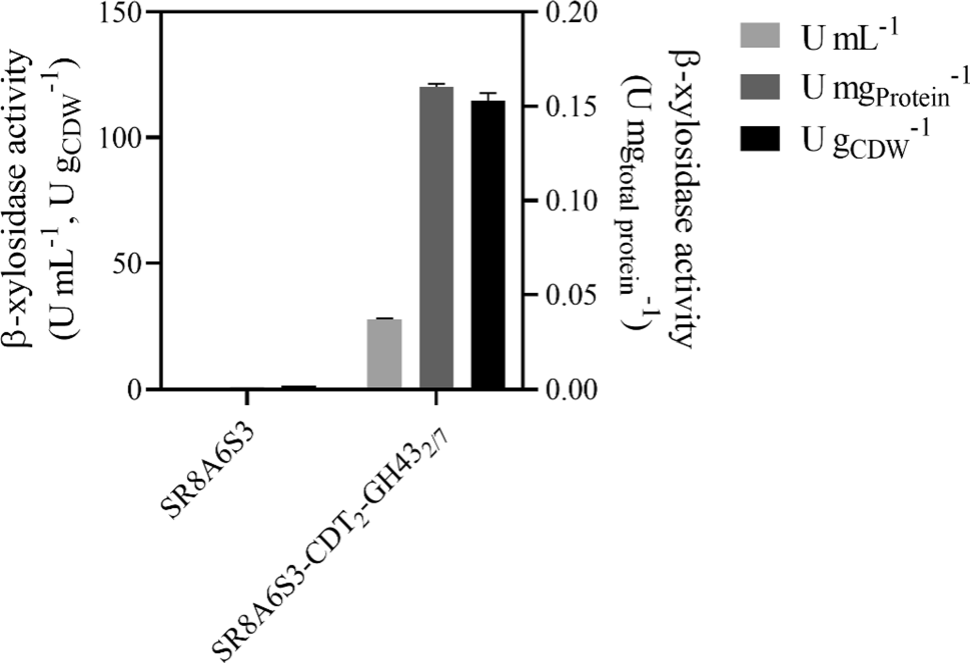
Intracellular β-xylosidase activity of SR8A6S3 and SR8A6S3-CDT-2-GH43-2/7 pellet extracts. The strains were cultured in YP-medium supplemented with 20 g L^−1^ glucose, 80 g L^−1^ xylose, and 8 g L^−1^ acetate) under microaerobic conditions until the late log phase. The intracellular β-xylosidase activity of GH43-2 and GH43-7 with pNPX as substrate were calculated relative to mg of protein and g of cell dry weight.

The β-xylosidase activity assay of GH43-2 and GH43-7 in cell extracts of SR8A6S3 and SR8A6S3-CDT-2-GH43-2/7 was determined with pNPX as substrate (Fig. 4). No β-xylosidase activity was detected in the control strain, which is consistent with the absence of both the GH43-2 and GH43-7 genes from its genome. On the other hand, the SR8A6S3-CDT-2-GH43-2/7 strain showed β-xylosidase activities of 27.74 U mL^−1^, or 114.53 U g_CDW_^−1^, or 0.160 U mg_Protein_^−1^. The expression of both GH43-2 and GH43-7 is essential for converting XOS into xylose, as the XR also acts as an XOS reductase, producing xylosyl-xylitol as a potential dead-end product, as first reported by Li et al.^25^. According to their work, despite the β-xylosidase GH43-7 having weak β-xylosidase activity, it rapidly cleaves xylosyl-xylitol into xylose and xylitol^25^.

Within the context of XOS-to-ethanol production, other fungal xylanases have also been functionally expressed in *S. cerevisiae*, for example: β-xylosidase from *Aspergillus oryzae* NiaD300 and xylanase II from *Trichoderma reesei* QM9414, which had activities in *S. cerevisiae* MT8-1 of 234 U g_CDW_^−1^ and 16 U g_CDW_^−1^, respectively^40^; β-xylosidase from *T. reesei* QM9414 demonstrated activity of 6 nmol min^−1^ mg_Protein_^−1^ in *S. cerevisiae* M4-D4^41^; Sakamoto et al.^42^ expressed an endoxylanase (*T. reesei*) and a β-xylosidase (*A. oryzae*) in *S. cerevisiae* MT8-1 and their activities were 41.2 U g_CDW_^−1^ and 16.8 U g_CDW_^−1^, respectively (Sakamoto et al.^42^); and most recently, endoxylanase from *T. reesei* QM6a was expressed in *S. cerevisiae* EBY100 giving activity of 1.197 U mg^−1^^43^.

### Fermentation of hydrolysed xylan by the engineered SR8A6S3-CDT-2-GH43-2/7 strain

**Figure 5:**
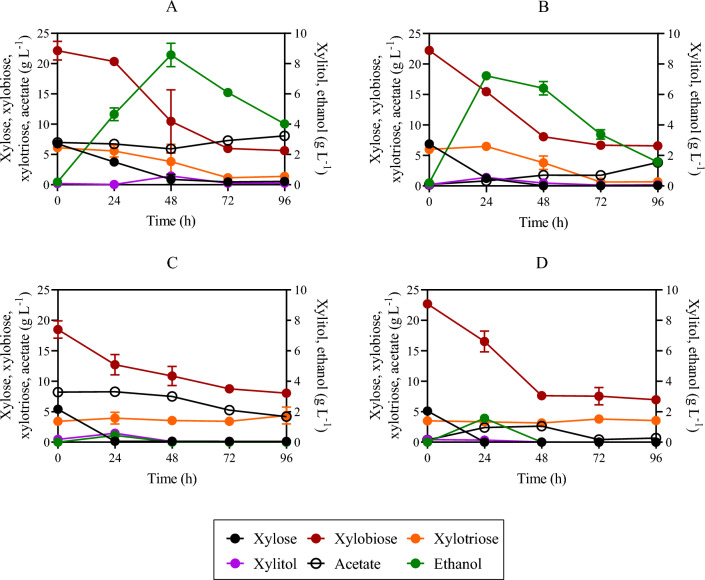
Fermentation profiles of SR8A6S3-CDT-2-GH43-2/7 (**A** and **B**) and SR8A6S3 (**C** and **D**) during batch cultivation in YPAXyl (YP medium containing hydrolysed xylan and acetate), (**A**) and (**C**), and YPXyl (YP medium containing hydrolysed xylan), (**B**) and (**D**). Cultivations were performed at 30 °C and 100 rpm with an initial OD_600_ of 10. Data are presented as the mean value and standard deviation of two independent biological replicates.

To evaluate XOS utilization, strain SR8A6S3-CDT-2-GH43-2/7 and the parental (control) strain SR8A6S3 were cultivated under micro-aerobic conditions at 30 °C, in a YP medium supplemented with hydrolysed xylan (YPXyl) and a mix of hydrolysed xylan plus acetate (YPAXyl) media. These media were designed to mimic a hemicellulosic hydrolysate, without the presence of inhibitory compounds, which can negatively influence yeast fermentations^13,44^. The engineered SR8A6S3-CDT-2-GH43-2/7 strain and its parental SR8A6S3 strain were cultivated in YPXy (Fig. 5B and D), and in YPAXyl (Fig. 5A and C) with an initial OD_600_ of 10. As expected, the engineered SR8A6S3-CDT_2_-GH43_2/7_ strain produced higher titres of ethanol than the parental SR8A6S3 strain under all test conditions.

In the medium, both X2 and X3 were the main carbon sources provided. X2 concentrations decreased during the growth of both the SR8A6S3-CDT-2-GH43-2/7 and SR8A6S3 strains (Fig. 5); however, SR8A6S3 did not express either heterologous xylanolytic enzymes or a XOS-transporter. One explanation could be that X2 entered the cell through a natural transport system in *S. cerevisiae* and was converted into the non-metabolizable xylosyl-xylitol compound by XR (xylose reductase), as observed previously by Li and colleagues^25^. It is important to note that *S. cerevisiae* can consume disaccharides such as maltose, sucrose, and trehalose, which are up taken through the action of membrane transporters^45^. The uptake of sucrose (disaccharide composed of glucose and fructose) can occur via the proton-symport (Mal11p)^46^. While trehalose (disaccharide composed of two glucose) can be taken up via Agt1p-mediated trehalose transport, followed by intracellular hydrolysis catalysed by trehalase *Nth1*. Furthermore, the *AGT1*/*MAL11* gene is controlled by the *MAL* system. Maltose is transported to the cytosol by an energy-dependent process coupled to the electrochemical proton gradient (Lagunas^45^).

Within the first 24 h of cultivation, SR8A6S3 depleted all xylose present in the medium (Fig. 5C and D) while SR8A6S3-CDT-2-GH43-2/7 spent more time fermenting xylose completely (Fig. 5A and B). At the same time, the doubly-engineered strain consumed 6.77 ± 0.03 g L^−1^ of X2, which represents 30% of the initial X2 concentration and 1.79 ± 1.08 g L^−1^ of X2 (8% of the initial X2 concentration) respectively, for the cultivations in YPXyl and YPAXyl. The presence of acetate changed the X2 consumption profile of SR8A6S3-CDT_2_-GH43_2/7_ (Fig. 5A). Intriguingly, the presence of X2 changed the acetate consumption profile of the parent strain, which consumed 4% of the initial acetate concentration up to 24 h, and 10% of the initial acetate concentration within first 48 h of cultivation (Fig. 5C). The slight acetate reduction between 24 and 48 h of cultivation might be affected by the oxidation of ethanol^47^, which peaked at 24 h (Fig. 5C). Concerning the X3 consumption profile, the SR8A6S3 parent strain barely metabolized X3 in either medium (Fig. 5C and D). Conversely, the engineered strain started to metabolize X3 after 24 h. The higher initial concentration of X2 than X3 probably interfered with X3 transportation. Instead, in 24–48 h, SR8A6S3-CDT-2-GH43-2/7 consumed 2.67 ± 0.85 g L^−1^ and 1.73 ± 1.32 g L^−1^ of X3 from YPXyl (Fig. 5B) and YPAXyl (Fig. 5A) cultivations, respectively. After 72 h of cultivation, no substantial decrease in X3 amount was observed for XOS-consuming strain cultivations.

The ethanol yield from SR8A6S3-CDT-2-GH43-2/7 was much higher than the control in both media (Table 2). Furthermore, after 96 h of cultivation, both strains consumed approximately the same amount of X2 in YPXyl (Fig. 5B and D), only strain SR8A6S3-CDT-2-GH43-2/7 appeared to ferment it to ethanol. Deletion of *gre3* and *sor1* delayed xylitol production by the SR8A6S3-CDT_2_-GH43_2/7_ strain. Interestingly, similar amounts of xylitol were observed for both control and engineered strains when cultured in YPAXyl, 0.59 ± 0.00 g L^−1^ and 0.57 ± 0.10 g L^−1^, respectively. However, this was only evident at 24 h of cultivation for SR8A6S3 and 48 h for SR8A6S3-CDT-2-GH43-2/7.

**Table 2:** Ethanol yield based solely on monomeric xylose of SR8A6S3-CDT-2 and SR8A6S3 strains under micro-aerobic cultivation at 30 °C in YP medium, supplemented with a mix of hemicellulosic hydrolysate plus xylose and acetate (YPXAH), hydrolysed xylan (YPXyl) or a mix of hydrolysed xylan plus acetate (YPAXyl) with varied initial OD_600_.

	Cultivation medium	Initial OD_600_	Y_ethanol_
SR8A6S3-CDT-2-GH43-2/7	YPXAH	1	0.50 ± 0.03
	YPXAH	20	0.58 ± 0.08
	YPXyl	10	1.24 ± 0.04
	YPAXyl	10	1.43 ± 0.05
SR8A6S3	YPXAH	1	0.33 ± 0.08
	YPXyl	10	0.31 ± 0.00
	YPAXyl	10	0.08 ± 0.00

Parameters: Y_ethanol_, ethanol yield on consumed monomeric xylose (g_ethanol_ g_xylose_^−1^).

### Fermentation of hemicellulosic hydrolysate by the engineered SR8A6S3-CDT-2-GH43-2/7 strain

**Figure 6:**
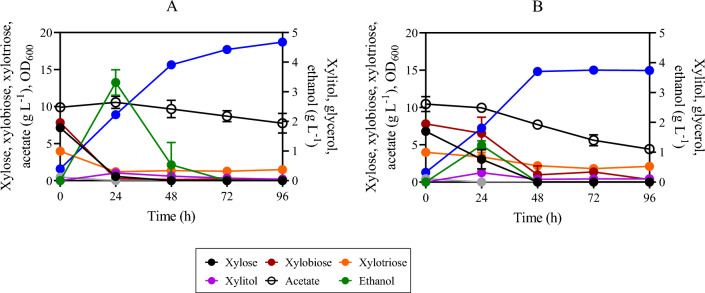
Fermentation profiles of SR8A6S3-CDT-2-GH43-2/7 (**A**) and SR8A6S3 (**B**) during batch cultivation in YPXAH (YP medium containing xylose, acetate, and hydrolysed hemicellulose). Cultivations were performed at 30 °C and 100 rpm with an initial OD_600_ of 1. Data are presented as mean values and standard deviations of two independent biological replicates.

Following successful cultivation in a simulated hemicellulose hydrolysate, strain SR8A6S3-CDT_2_-GH43_2/7_ was cultivated under micro-aerobic conditions in a YP medium supplemented with an authentic XOS-rich hemicellulosic hydrolysate^48^ (Fig. 6A and B). This mimics the context of a lignocellulosic biorefinery, which makes full use of hemicellulose. The breakdown of hemicellulose, which is acetylated^13^, releases highly toxic acetate; reducing the fermentative performance of *S. cerevisiae*^21,25^. The SR8A6S3 strain was previously engineered through an optimized expression of AADH and ACS in the acetate reduction pathway, enabling acetate conversion into ethanol by the optimized strain^22^. Therefore, the current study tested whether the acetate reduction pathway could operate simultaneously with XOS fermentation, as a means to augment ethanol yield from the lignocellulosic hydrolysate.

Under this condition, we observed that xylose, X2, and X3 presented similar consumption profiles in the XOS-consuming strain cultivation. These carbon sources were primarily consumed before 24 h of cultivation. The latest engineered strain consumed 6.57 ± 0.28 g L^−1^ of xylose, which represents 92% of the initial xylose concentration, and 7.57 ± 0.08 g L^−1^ of X2, which represents 97% of the initial X2 concentration, and 2.76 ± 0.14 g L^−1^ of X3 (69% of the initial X3 concentration). Conversely, during the same period, the parent strain consumed only 3.73 ± 0.94 g L^−1^ of xylose (55% of the initial xylose concentration), and 16% and 15.5% of the initial X2 and X3 concentrations, respectively. It is worth pointing out that, as aforementioned, SR8A6S3 did not express either heterologous xylanolytic enzymes or an XOS-transporter; therefore, the uptake of XOS probably occurs through the action of membrane transporters that carry out disaccharide transport.

Although we observed a decrease in X2 and X3 amounts in cultivations with the control strain, only cultivations with the XOS-consuming strain (SR8A6S3-CDT-2-GH43-2/7) was ethanol accumulation consistent with X2 fermentation; i.e., conversion of X2 into ethanol (Fig. 6A). The SR8A6S3-CDT-2-GH43-2/7 and SR8A6S3-CDT_2_ strains achieved the highest ethanol concentration at 24 h of cultivation, 3.78 ± 0.53 g L^−1^ and 1.23 ± 0.10 g L^−1^, respectively. In other terms, the newest engineered strain achieved an ethanol yield of 0.50 ± 0.03 g _ethanol_ g _consumed xylose_^−1^, and the control strain ethanol yield was 0.33 ± 0.08 g _ethanol_ g _consumed xylose_^−1^ (Table 2). Interestingly, the ethanol peak did not follow xylose exhaustion in the control cultivation (Fig. 6B), as happened with the engineered strain cultivation (Fig. 6A).

Acetate consumption was not observed in both SR8A6S3 and SR8A6S3-CDT-2-GH43-2/7 cultivations within the first 24 h of cultivation. These results might indicate that transportation of X2 and X3 might result in changes of the NADH / NAD^+^ and ATP balance, which impaired the acetate consumption profile. In a previous study, Zhang et al.^22^ highlighted that three major factors might limit the metabolic fluxes of the acetate reduction pathway, these include the intracellular ATP levels, NADH levels, and the activities of key enzymes (ACS and AADH) being the last major limiting factor among them. In this study, the expression of key enzymes was not modified through genetic intervention. Between 24 and 96 h of cultivation, acetate was reduced by 25% and 53% for the XOS-consuming and control strains, respectively. The acetate profile seems to be a combination of acetate consumption and acetate production, resulting from ethanol oxidation^47^. The lesser change in acetate consumption profile for SR8A6S3-CDT-2-GH43-2/7 than SR8A6S3 might have resulted from the higher amount of ethanol produced by this strain, which could be converted into acetate after exhaustion of the sugars^47^. Hence, it appears to use less acetate. Regarding xylitol production, the SR8A6S3-CDT-2-GH43-2/7 strain produced a lower xylitol yield, 0.041 ± 0.01 g _xylitol_ g _consumed xylose_^−1^, than the control cultivation, in which the yield was 0.083 ± 0.01 g _xylitol_ g _consumed xylose_^−1^.

To evaluate the improvement obtained through the introduction of the XOS-consumption pathway in the SR8A6S3 strain, ethanol yield based on consumed monomeric xylose was calculated for each condition (Table 2). This was calculated to highlight that ethanol was also produced by consumption of XOS in addition to monomeric xylose consumption, since stochiometric maximal conversion of xylose into ethanol is around 0.51 g _ethanol_ g _xylose_^-1^. The ethanol yield of the SR8A6S3-CDT-2-GH43-2/7 strain increased substantially as compared to the SR8A6S3 strain. This substantial yield increase is very likely due to the additional conversion of XOS to ethanol.

Lignocellulose-derived ethanol provides environmental and economic benefits besides being a promising industry in the expected transition from fossil fuels to renewable energy^13^. Hemicellulosic-derived sugar comprises 15–35% of lignocellulosic biomass, representing a large source of renewable material that is available at a low cost^6,13,49^. Engineered strains able to consume XOS derived from hemicellulose via intracellular hydrolysis represent a potential benefit for bioethanol production; since these strains would have a competitive advantage concerning other microorganisms, such as contaminating bacteria and wild *Saccharomyces* and non-*Saccharomyces* species that are expected to be unable to utilize XOS as a carbon source^7,50^.

## Conclusions

Xylose metabolism into ethanol in *S cerevisiae* SR8A6S3 is metabolically inefficient due to the production of xylitol. In this study we have integrated genes necessary to create a XOS-consumption pathway into two xylitol-production-related genes, *SOR1* and *GRE3*. The resulting strains, SR8A6S3-CDT-2 and SR8A6S3-CDT-2-GH43-2/7, which are *sor1*Δ and *sor1*Δ-*gre3*Δ, respectively, showed a reduction in xylitol production and improvement in ethanol yield when compared with their parental strain SR8A6S3 in YPDXA cultivations under both micro-aerobic and anaerobic conditions. However, this coincided with a reduced rate of xylose metabolism, implying that there is scope for improvement in overall flux from xylose to ethanol. SR8A6S3-CDT_2_-GH43_2/7_ was able to ferment X2 and X3 efficiently for ethanol production, and achieved the highest apparent ethanol yield (based only on the content of monomeric xylose) of 1.43 ± 0.05 g ethanol g xylose^-1^ (64% higher than theoretical ethanol yield) in YP supplemented with hydrolysed xylan and acetate. When grown on a medium containing hemicellulosic hydrolysate with low monomeric xylose content, fermentation of X2 and X3 was poor, but this was dramatically improved with the addition of monomeric xylose. This, and other evidence which shows that X2 and X3 metabolism slows down once the monomeric carbohydrates have been depleted, suggests that the latter are required to provide the energy demands of the former (e.g. for enzyme biosynthesis). While there is clearly room for further improvement, this demonstrates that a XOS fraction generated by simple hydrothermal/steam explosion pre-treatment of lignocellulosic agricultural residues, without any subsequent enzymatic hydrolysis, is a potential resource for renewable biofuel production using a XOS-utilising yeast.

## Materials and methods

### Strains and media

*E. coli* strain DH5α was used for the construction and propagation of plasmids. *E. coli* was cultured in Lysogeny Broth (LB) medium (5 g L^−1^ yeast extract, 10 g L^−1^ tryptone, and 10 g L^−1^ NaCl) at 37 °C, and 100 µg mL^−1^ ampicillin (LBA) was added for selection when required. All engineered *S. cerevisiae* strains used and constructed in this work are summarized in Table 3. Yeast strains transformed with plasmids containing antibiotics were propagated on YPD plates supplemented with the plasmid corresponding antibiotics, such as clonNAT (100 µg mL^−1^), geneticin G418 (200 µg mL^−1^), and hygromycin B (200 µg mL^−1^). The SR8A6S3-CDT_2_ strain was generated by integrating the CDT-2 transporter overexpressing gene cassette into the *SOR1* locus of the SR8A6S3 genome. To construct an XOS-utilizing strain the P_*GAP*_*-*GH43-7-T_*CYC1*_-P_*CCW12*_-GH43-2-T_*CYC1*_ was integrated at the *GRE3* locus of SR8A6S3-CDT-2, yielding strain SR8A6S3-CDT-2-GH43-2/7.

**Table 3:** The yeast strains used in this study.

Strain	Description	References
SR8	Efficient xylose-utilizing strain engineered from strain D452-2 (evolved strain of D452-2 *leu2::LEU2*_pYS10, *ura3::URA3_*pRS6-X123, *his1::HIS1_*pRS3-X23, and *ald6::AUR1-C* pAUR_d_*ALD6*)	^51^
SR8A6S3	SR8 expressing three copies of codon optimised AADH gene (coded by *E. coli adhE* gene) overexpression cassette and three copies of mutant *Salmonella* ACS gene overexpression cassette	^22^
SR8-XD	SR8 expressing one copy of CDT2, GH43-2, and GH43-7 overexpression cassette	^52^
SR8A6S3-CDT-2	SR8A6S3 expressing one copy of CDT2 overexpression cassette (*sor1*::CDT-2)	This work
SR8A6S3-CDT-2-GH43-2/7	SR8A6S3-CDT-2 expressing one copy of GH43-2 and GH43-7 overexpression cassette (*sor1*::CDT-2 and *gre3*::GH43-2/7)	This work

### Plasmids and strain construction

All plasmids and primers in this work are summarized in Tables 4 and 5, respectively. The guide RNA (gRNA) plasmids (Table 6), gRNA-sor-K and gRNA-gre-K, were amplified from Cas9-NAT by using primer pairs DPO_089 and DPO-090, DPO_087 and DPO_088 carrying a 20 bp PAM sequence for *SOR1* and *GRE3 loci*, respectively. The gRNAs were predicted by the website^53^. All gRNA sequences are listed in Table 5**.**

**Table 4:** Plasmids used in this study.

Plasmids	Description	References
pRS42K	pRS42K, Kanamycin resistance gene	^55^
p426GPD	pRS426-P_*TDH3*_-T_*CYC1*_	Addgene (#14156)
Cas9-NAT	P414-P_*TEF1*_-Cas9-T_*CYC1*_-NAT1	Addgene (#64329)
p426-CDT-2	pRS425-P_*PGK1*_-CDT-2-T_*CYC1*_	^56^
gRNA-sor-K	pRS42K carrying *SOR1* disruption gRNA cassette	This work
gRNA-gre-K	pRS42K carrying *GRE3* disruption gRNA cassette	This work
p426-GH43-2/7	pRS426-P_*TDH3*_-GH43-7-T_*CYC1*_-P_*CCW12*_-GH43-2-T_*CYC1*_	This work

**Table 5:** Primers used in this study.

Name	Sequence (5′–3′)
DPO_059	ATGCCCCTCGTCAAGAACCCCATCCTCCCCGGCTTCAATC
DPO_063	TTACTTCCCAGCCGGCTGCTTTTCCCCACAAATCTTCCCCTCTTCA
DPO_064	CCGGGCTGCAGGAATTCGAT
DPO_065	GGGATCCACTAGTTCTAGAA
DPO_062	CCAGAACTTAGTTTCGACGGATTCTAGAACTAGTGGATCCCATGCCCCTCGTCAAGAACC
DPO_074	GACGGTATCGATAAGCTTGATATCGAATTCCTGCAGCCCGGTTACTTCCCAGCCGGCTGC
DPO_057	GTAATATAAATCGTAAAGGAAAATTGGAAATTTTTTAAAGGTAATACGACTCACTATAGG
DPO_058	TTGTTCATATCGTCGTTGAGTATGGATTTTACTGGCTGGAAATTAACCCTCACTAAAGGG
DPO_081	AATCAACAAGAAAAAATACTAAAAAAAAAAATTGAAAAATGTAAAACGACGGCCAGT
DPO_082	TATATATGGACATGAACCAGTGCCGAAAAGTATTCACTTTACAGGAAACAGCTATGAC
DPO_089	TGTGTCGAACCCTTATCAGTGTTTTAGAGCTAGAAATAGCAAG
DPO_090	ACTGATAAGGGTTCGACACAGATCATTTATCTTTCACTGCGGA
DPO_083	CCGGTCTCGTATCTCCTTT
DPO_084	CTATCAACTGGAAGTAATGCG
DPO_069	GGGGGCCTATCAAGTAAATTACTCCTGGT
DPO_070	GTTCAGATTCACTTCTTGATATTTCC
DPO_087	TCCTCAATCATTCATTGAGAGTTTTAGAGCTAGAAATAGCAAG
DPO_088	TCTCAATGAATGATTGAGGAGATCATTTATCTTTCACTGCGGA

**Table 6:** gRNA used in this study.

gRNA (5′– 3′)	Insertion locus	Plasmid	Reference
TGTGTCGAACCCTTATCAGT	*SOR1*	gRNA-sor-K	This study
TCCTCAATCATTCATTGAGA	*GRE3*	gRNA-gre-K	This study

For genomic integration of CDT-2 through CRISPR-Cas9-based integration in the *SOR1* gene site of SR8A6S3, CDT-2 donor DNA was amplified from plasmid pRS426-CDT2 using a primer pair DPO_081 and DPO_082. Transformants with CDT-2 integration were identified by PCR using primers DPO_083 and DPO_084, and the resulting strain was designated as the SR8A6S3-CDT-2 (Table 3). The PCR reaction was performed using 1.25 µL forward primer, 1.25 µL reverse primer, 1 µL DNA sample, 12.5 µL Phusion high-fidelity DNA polymerase master mix with HF buffer (New England BioLabs), and 9 µL of nuclease-free water.

To generate transformant strains expressing the GH43-7_GH32-2 gene cassette, the sequence GH43-7-T_*CYC1*_-P_*CCW12*_-GH43-2 was amplified from the genomic DNA of the XOS-consuming strain, SR8-XD (Table 5). Firstly, SR8-XD genomic DNA was prepared with the Rapid Yeast Genomic DNA Extraction Kit (Bio Basic Inc., Markham Ontario, CA) and quantified by NanoDrop ND-1000. The primer pair of DPO_059 and DPO_063 were used to amplify the GH43-7-T_*CYC1*_-P_*CCW12*_-GH43-2 gene sequence. The GH43-7-T_*CYC1*_-P_*CCW12*_-GH43-2 PCR product was amplified again using the primer pair of DPO_062 and DPO_074, which has homology with plasmid p426GPD. Similarly, the plasmid p426GPD was amplified using a primer pair of DPO_064 and DPO_065. PCR was performed using 1.25 µL forward primer, 1.25 µL reverse primer, 1 µL DNA sample, 12.5 µL of Phusion high-fidelity DNA polymerase master mix with HF buffer (New England BioLabs), and 9 µL of nuclease-free water. Both the linear sequences were transformed into competent *E. coli* DH5α to form the plasmid p426-GH43-2/7^54^ (Table 5). P_*TDH3*_-GH43-7-T_*CYC1*_-P_*CCW12*_-GH43-2-T_*CYC1*_ donor DNA was amplified from plasmid p426-GH43_2/7_ using the primer pair DPO_057 and DPO_058.

Transformation of yeast cells was carried out by the polyethylene glycol (PEG)-LiAc method^57^. One microgram of DNA was used for Cas9 or gRNA plasmid transformation, 1.5 µg of donor DNA was used for homologous recombination. Correct integration was confirmed by PCR using primers DPO_069 and DPO_070. The recombinant strain was designated as SR8A6S3-CDT-2-GH43-2/7 (Table 3).

### Enzyme activity assay and protein quantification

SR8A6S3-CDT-2-GH34-2/7 and SR8A6S3 were grown in 22 mL of yeast extract‐peptone (YP) medium (10 g L^−1^ yeast extract, 20 g L^−1^ peptone) containing 2% glucose, 8% xylose, and 0.8% acetate (YPDXA) until late log phase, before harvest by centrifugation. Yeast cell pellets, 0.24 g for SR8A6S3-CDT-2-GH34-2/7 and 0.21 g for SR8A6S3, were resuspended in buffer containing 0.1 mM CaCl_2_, 50 mM Tris–HCl, 100 mM NaCl, 1 mM DTT, 0.1% Triton X, at pH 7.4, and 0.1 mM PMSF (Thermo Fisher Scientific). The cells were disrupted by agitation using 1 g glass beads and an ultrasonic bath at 40% amplitude for 5 min on ice. The resulting lysates were centrifugated at 14,000 × g for 20 min at 4 °C, and the clarified supernatant was used as an enzyme source for β-xylosidase assays.

β-xylosidase activity was measured according to Tramontina et al.^58^. Briefly, 30 µL of the clarified supernatant and 50 µL of 5 mM ρ-Nitrophenyl-β-d-xylopyranoside (pNPX) solution were added to 20 µL of reaction buffer (250 mM MES, and 5 mM CaCl_2_, pH 7); which was then incubated at 30 °C for 60 min for the enzyme reaction. The reaction was stopped by adding 100 µL of 2 M Na_2_CO_3_ and the amount of ρ-Nitrophenol produced was estimated spectrophotometrically at a wavelength of 405 nm; and the absorbance converted to concentration using a standard curve. One unit of enzyme activity was defined as the amount of enzyme catalysing the hydrolysis of 1 µmol pNPX per minute in 1 mL of yeast intracellular lysate (μmol mL^−1^ min^−1^) “U mL^−1^”, or per mg of total lysate protein (μmol mg^−1^ min^−1^) “U mg^-1^”, or per gram of cells (μmol g_CDW_^−1^ min^−1^) under the described assay conditions. The protein concentrations of each sample were determined using the Bradford dye method^59^.

### Fermentation and analytical methods

Anaerobic batch fermentation experiments were performed in 100 mL serum bottles with 30 mL fermentation media. Serum bottles were sealed with a butyl rubber stopper and then flushed with nitrogen gas, which had been passed through a heated, reduced copper column to remove traces of oxygen. Micro-aerobic batch fermentation experiments were performed in a 125 mL Erlenmeyer flask with 30 mL of fermentation media. Both anaerobic and micro-aerobic cultures were incubated in a rotary shaker at 100 rpm at 30 °C.

For all cultivations, yeasts were pre-grown in yeast extract‐peptone (YP) medium (10 g L^−1^ yeast extract, 20 g L^−1^ peptone) supplemented with 20 g L^−1^ glucose, and harvested by centrifugation at 3,134 × g, at 4 °C for 5 min, and washed three times with sterile distilled water. Washed yeast cells were inoculated in serum bottles or Erlenmeyer flasks containing either: YP supplemented with a mixture of glucose, xylose, and acetate (YPDXA); hemicellulosic hydrolysate (YPH); hemicellulosic hydrolysate, xylose and acetate (YPXAH); hydrolysed xylan (YPXy); and hydrolysed xylan and acetate (YPAXy). Initial cell concentration varied according to the cultivation, OD_600_ was 1 or 10. Xylan hydrolysis was carried out according to^60^. The hemicellulosic hydrolysate from sugar cane straw was obtained by a two-stage procedure: mild acetylation at 60 °C, 30 min, 0.8% (w w^−1^) of NaOH and 10% (w w^-1^) of solids, followed by hydrothermal pre-treatment at 190 °C, 20 min, 10% (w w^−1^) of solids. The hemicellulosic hydrolysate obtained after the second step was enzymatically treated with a GH11 from *Neocallimastix patriciarum* (Megazyme® Ireland), as detailed elsewhere^48^. Afterwards, the hemicellulosic hydrolysate rich in XOS was concentrated, approximately five-fold, in a rotary vacuum evaporator. Table 7 shows the chemical composition of the XOS-rich hemicellulosic hydrolysate.

**Table 7:** Chemical composition of the XOS-rich hemicellulosic hydrolysate after treatment with an endoxylanase GH11 and concentration.

Component	AA	FA	FT	AR	AOS	GOS	Xyl	HMF	FL	XOS
Concentration (g L^−1^)	0.77	2.51	6.40	3.93	2.45	6.91	2.74	0.07	0.01	49.67

AA, acetic acid; FA, formic acid; FT, total phenolics; AR, arabinose; AOS, arabino-oligosaccharides; GOS, gluco-oligosaccharides; Xyl, xylose; HMF, hydroxymethylfurfural; FL, furfural; XOS, total xylo-oligosaccharides.

Samples were taken using a syringe and needle from serum bottles, or manual single-channel pipette (Gilson, USA) from Erlenmeyer flasks, at appropriate intervals to measure cell growth and metabolites concentrations. Cell growth was monitored as the optical density at 600 nm (OD_600_), measured using a UV–visible Spectrophotometer (Biomate 5). The samples were centrifuged at 14,000 × g for 10 min and supernatants diluted appropriately for the determination of glucose, xylose, xylitol, glycerol, succinate, acetic acid, and ethanol by high-performance liquid chromatography (HPLC, Agilent Technologies 1200 Series); equipped with a refractive index detector (RID). Chromatography was done on a Rezex ROA-Organic Acid H+ (8%) column (Phenomenex Inc., Torrance, CA) maintained at 60 °C, with 0.005 N H_2_SO_4_ as eluent at a flow rate of 0.6 mL min^−1^. Analyte concentrations were determined by using the RID detector.

### Xylo-oligosaccharide quantification

The enzymatic products were analysed by high-performance anion-exchange chromatography with pulsed amperometry detection (HPAEC–PAD), to detect xylose and XOS produced by the xylanase enzymes. Separation was performed using a Dionex ICS-3000 instrument (Thermo Fisher Scientific, Sunnyvale, CA, USA) with a CarboPac PA100 column (4 × 250 mm) and CarboPac PA100 guard column (4 × 50 mm), and eluted with a linear gradient of A (NaOH 500 mM) and B (NaOAc 500 mM, and NaOH 80 mM). The gradient program was 15% of A and 2% of B for 0–10 min, followed by 15–50% of A and 2–20% of B from 10–20 min, with a flow rate of 1.0 mL min^−1^. The integrated peak areas were converted to concentrations based on standards (× 1 to × 6).

### Supplementary Information

Supplementary Figures.

## Data Availability

All data generated or analysed during this study are included in this manuscript as supplementary information files.
