# Supplementary material for: Metabolic engineering of Saccharomyces cerevisiae for second-generation ethanol production from xylo-oligosaccharides and acetate
Source: Sci Rep. 2023 Nov 6;13:19182. doi: 10.1038/s41598-023-46293-8 (PMC10628280; doi:10.1038/s41598-023-46293-8)
Supplement: Supplementary file 1 — Supplementary Figures. [file 41598_2023_46293_MOESM1_ESM.pdf]

Supplementary information

Metabolic engineering of *Saccharomyces cerevisiae* for second-generation ethanol production from xylo-oligosaccharides and acetate

Dielle Pierotti Procópio[1], Jae Won Lee[2,3], Jonghyeok Shin[2,3], Robson Tramontina[4,5], Patrícia Felix Ávila[6], Lívia Beatriz Brenelli[7], Fabio Márcio Squina[5], André Damasio[4], Sarita Cândida Rabelo[8], Rosana Goldbeck[6], Telma Teixeira Franco[7,9], David Leak[10], Yong-Su Jin[2,3], Thiago Olitta Basso[1,*]

[1]Department of Chemical Engineering, Escola Politécnica, Universidade de São Paulo (USP), São Paulo, SP 05508-010, Brazil

[2]DOE Centre for Advanced Bioenergy and Bioproducts Innovation, University of Illinois at Urbana-Champaign, Urbana, IL 61801, USA

[3]Department of Food Science and Nutrition, University of Illinois at Urbana-Champaign (UIUC), Urbana, IL 61801, USA

[4]Department of Biochemistry and Tissue Biology, Institute of Biology, University of Campinas (UNICAMP), Campinas, SP 13083-862, Brazil

[5]Environment and Technological Processes Program, University of Sorocaba (UNISO), Sorocaba, SP 18023-000, Brazil

[6]School of Food Engineering, University of Campinas (UNICAMP), Campinas, SP 13083-862, Brazil

[7]Interdisciplinary Center of Energy Planning, University of Campinas (UNICAMP), Campinas, SP 13083-896, Brazil

[8]Departament of Bioprocesses and Biotechnology, School of Agriculture, Sao Paulo State University (UNESP), Botucatu, SP 18618-687, Brazil

[9]School of Chemical Engineering, University of Campinas (UNICAMP), Campinas, SP 13083-852, Brazil

[10]Department of Biology and Biochemistry, University of Bath, Claverton Down, Bath BA2 7AY, UK

***Correspondence:**

Thiago Olitta Basso

thiagobasso@usp.br

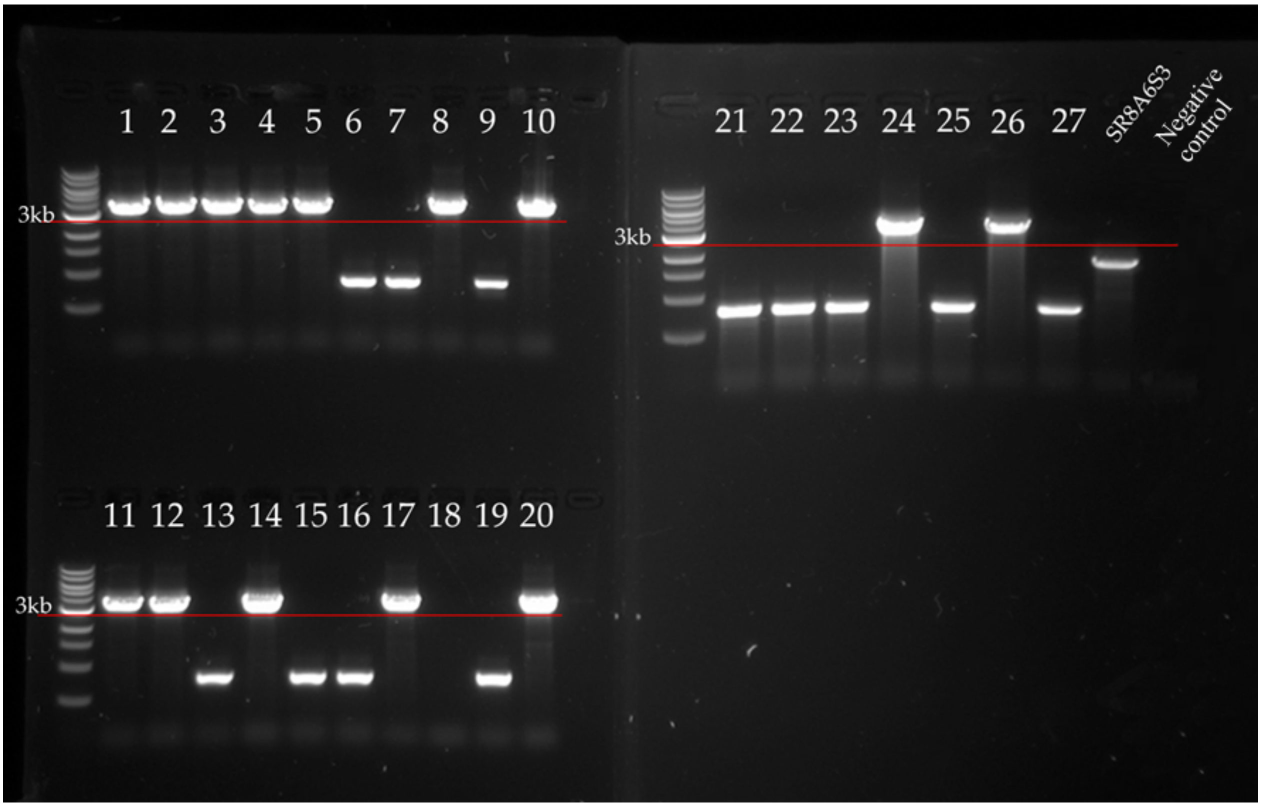

Fig. S1. Colony PCR products were analyzed using an agarose gel of 0.8% from positive control plate colonies. *sor1*Δ::$_P$*PGK1-CDT-2-*$_T$*CYC1* mutant cells present a band of 3.4 kb (1, 2, 3, 4, 5, 8, 10, 11, 12, 14, 17, 20, 24, 26), a deletion of the sor1 gene was observed in 6, 7, 9, 13, 15, 16, 19, 21, 22, 23, 25, 27 which band present 0.8 kb. The SR8A6S3 band represents the positive PCR control, of which the band has 1.8 kb. The last band represents the negative PCR control.

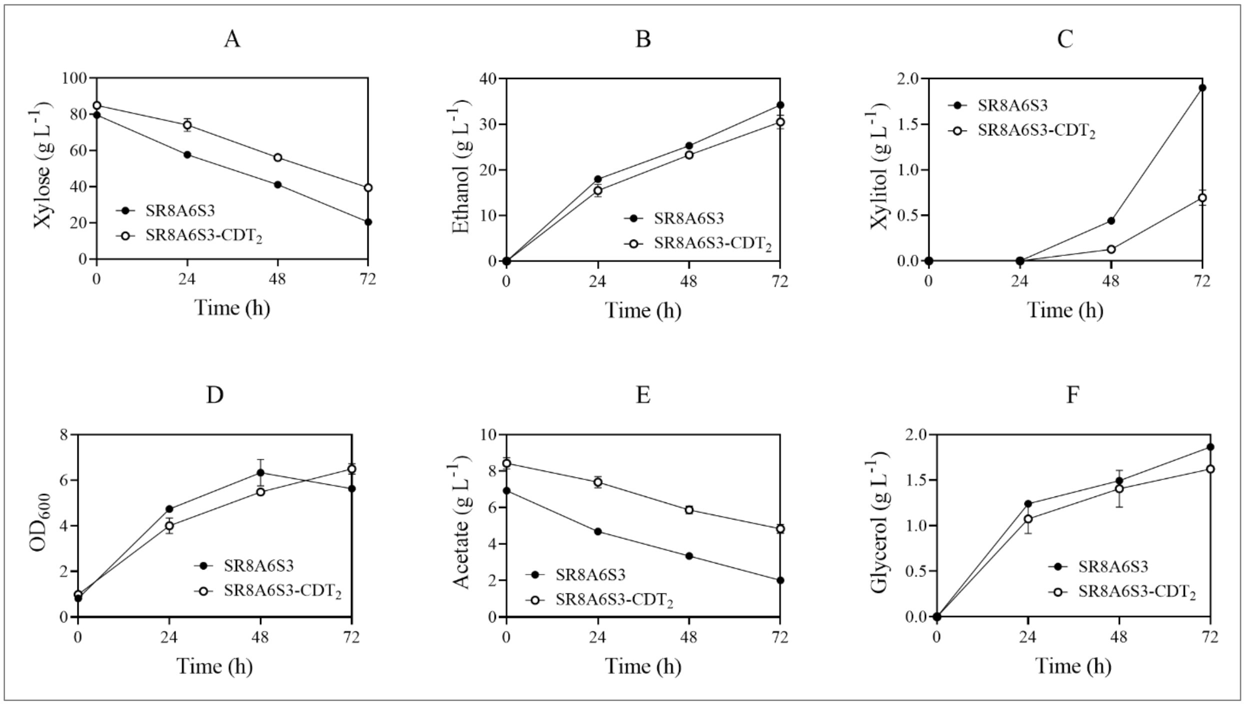

Fig. S2. Comparison of xylose (A) and acetate (E) consumption, ethanol (B), xylitol (C), OD_{600} (D), and glycerol (F) production of two xylose-acetate-assimilating strains, SR8A6S3-CDT_2 and SR8A6S3, in YP media containing 20 g L^{-1} glucose, 80 g L^{-1} xylose, and 8 g L^{-1} acetate under anaerobic condition. An initial OD_{600} was adjusted to 1. The figure illustrates the means of triplicate experiments for each strain.

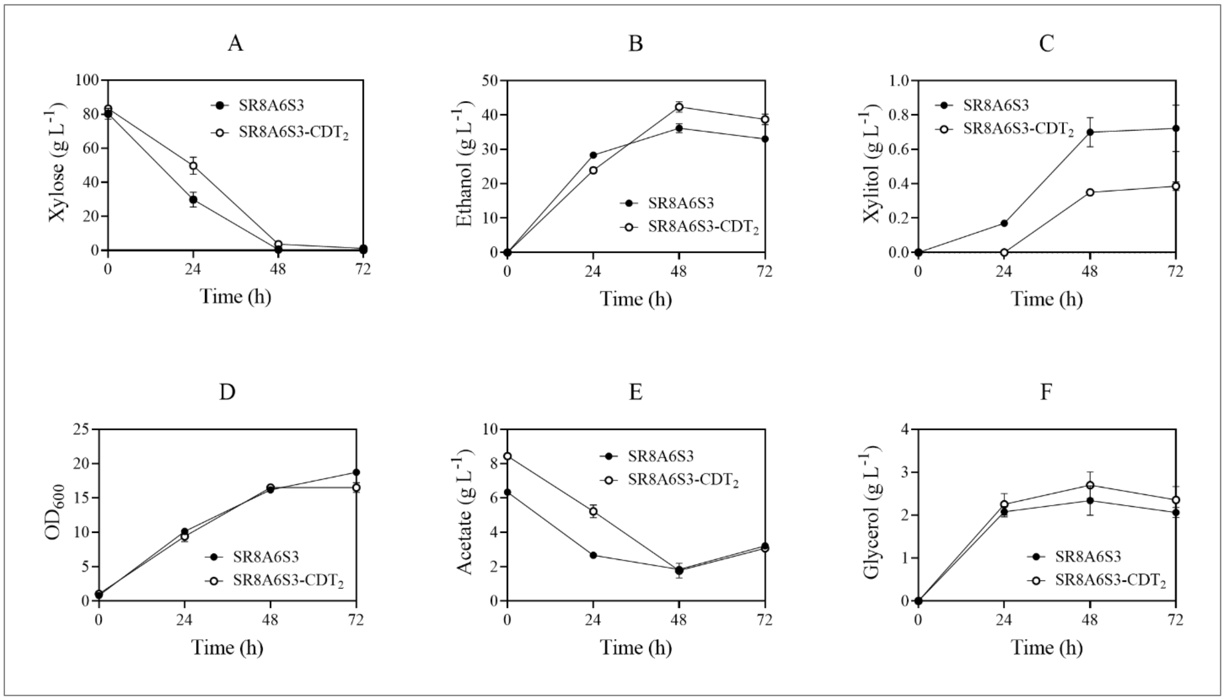

Fig. S3. Comparison of xylose (A) and acetate (E) consumption, ethanol (B), xylitol (C), OD_{600} (D), and glycerol (F) production of two xylose-acetate-assimilating strains, SR8A6S3-CDT_2 and SR8A6S3, in YP media containing 20 g L^{-1} glucose, 80 g L^{-1} xylose, and 8 g L^{-1} acetate under micro-aerobic condition. An initial OD_{600} was adjusted to 1. The figure illustrates the means of triplicate experiments of each strain.

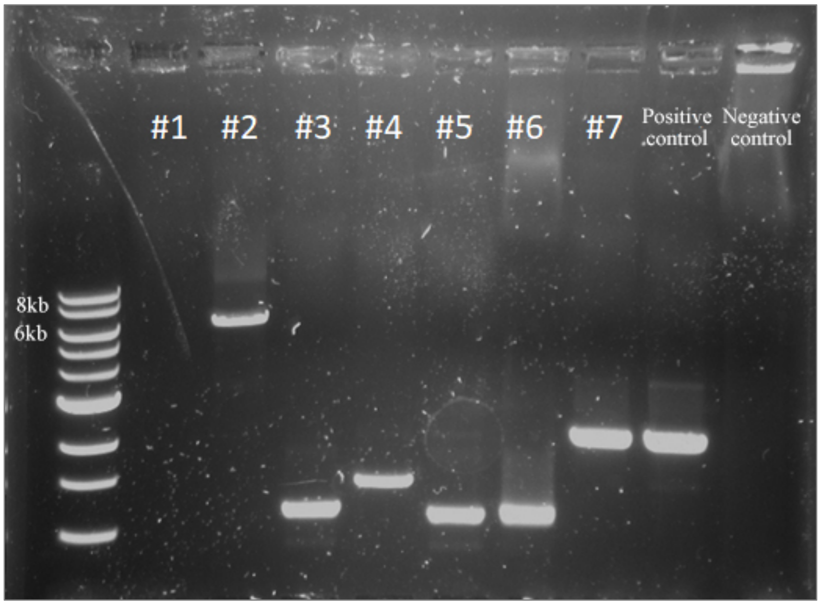

Fig. S4. Colony PCR products analyzed using an E-Gel 0.8% agarose from positive control plate colonies. *gre3*Δ:: $_{P}$TDH3-GH43-7-$_{T}$CYC- $_{P}$CCW12-GH43-2-$_{T}$CYC1 mutant cells present a band of 7 kb (colony 2), The positive PCR control band has 1.5 kb. The last band represents the negative PCR control.

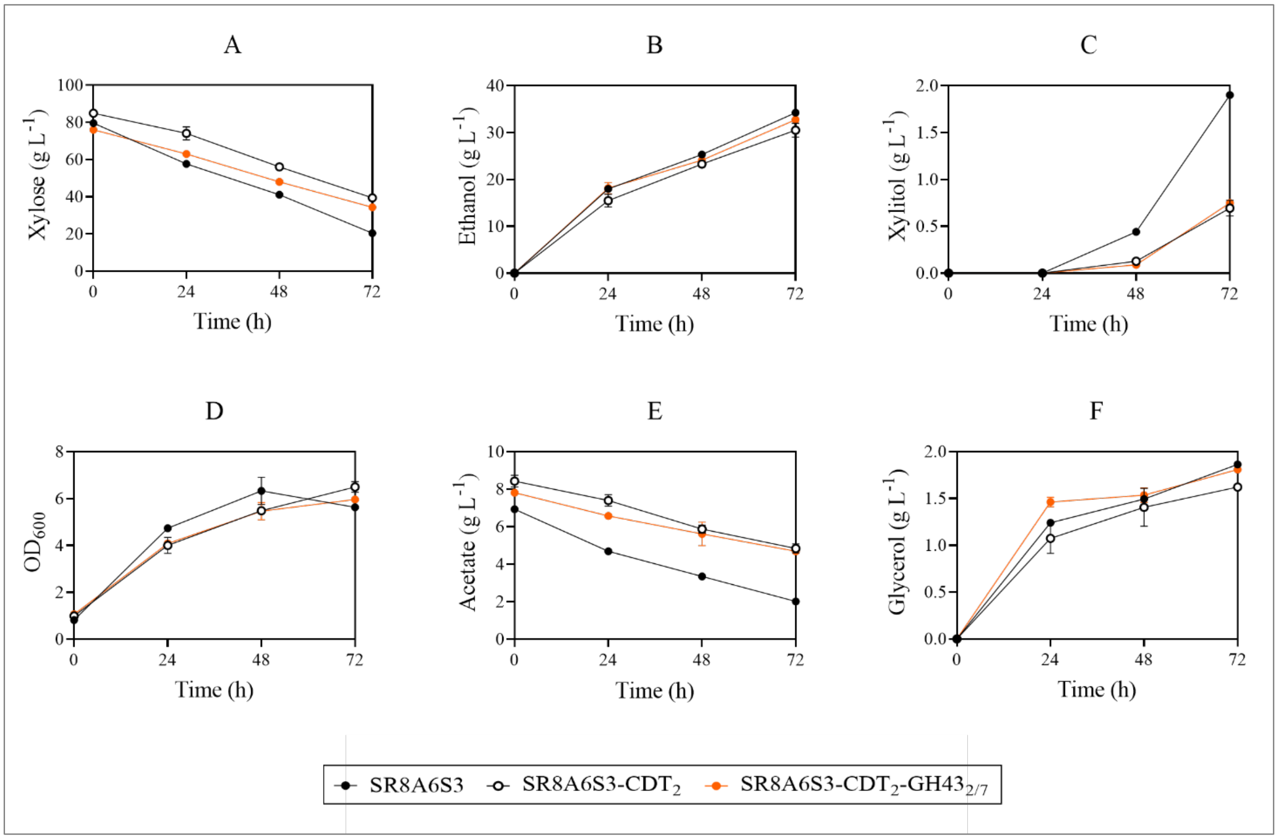

Fig. S5. Comparison of xylose (A) and acetate (E) consumption, ethanol (B), xylitol (C), OD_{600} (D), and glycerol (F) production of three xylose-acetate-assimilating strains, SR8A6S3, SR8A6S3-CDT_2, and SR8A6S3-CDT_2-$GH43_{2/7}$ cultivated in YP media containing 20 g L^{-1} glucose, 80 g L^{-1} xylose, and 8 g L^{-1} acetate under anaerobic condition. An initial OD_{600} was adjusted to 1. The figure illustrates the means of triplicate experiments of each strain.

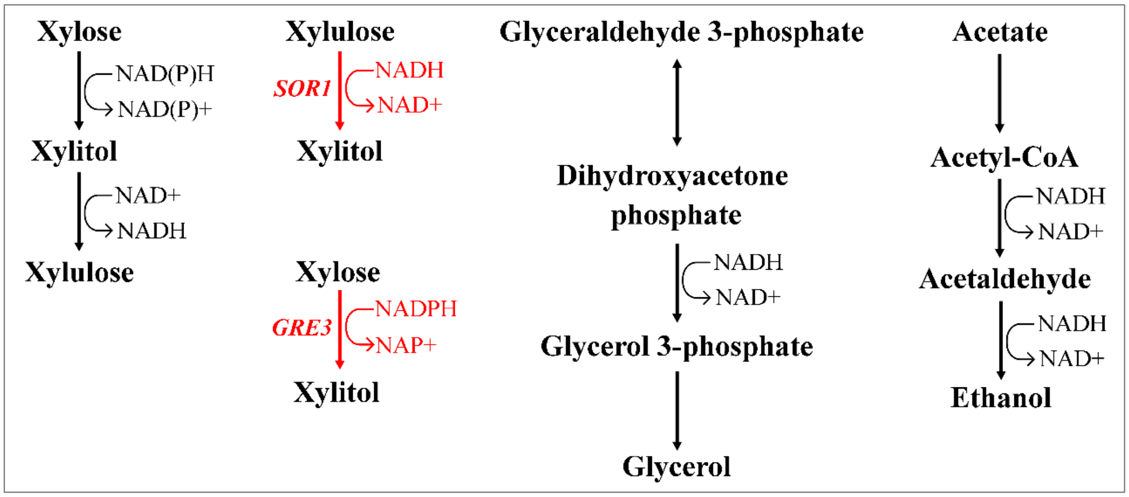

Fig. S6. Target metabolites were analyzed for comparison of fermentation profiles of the SR8A6S3-CDT$_2$-GH43$_{2/7}$ and SR8A6S3. The red arrow represents deleted gene/route.

NADH-linked acetylating acetaldehyde dehydrogenase (AADH)

acetyl-CoA synthetase (ACS

codon-optimized acetaldehyde dehydrogenase - *adhE* (CO_*adhE*)

mutated acetyl-CoA synthetase - *ACS* (*ACS*Opt*)

2G - second-generation

optimized minimum medium (oMM)

glycoside hydrolases (GH)

Pentose phosphate pathway - PPP

Xylobiose - X2

Xylotriose - X3
